# Addressing Racial Disparities in the Hypertensive Disorders in Pregnancy: A Plan for Action from the Preeclampsia Foundation’s Racial Disparities Task Force

**DOI:** 10.1007/s40615-024-02126-6

**Published:** 2024-08-26

**Authors:** Cornelia R. Graves, Tabassum Firoz, Skylar N. Smith, Natalie Hernandez, Shaconna Haley, Kim Smith, Robyn D’Oria, Ann C. Celi

**Affiliations:** 1 Tennessee Maternal Fetal Medicine, University of Tennessee Health Science Center, 201 23rd Ave., Nashville, TN 37203 USA; 2 Department of Medicine, Yale New Haven Health- Bridgeport Hospital, Bridgeport, CT USA; 3 Division of General Medicine and Primary Care, Department of Medicine, Brigham and Women’s Hospital, Boston, MA USA; 4 Center for Maternal Health Equity, Community Health and Preventative Medicine, Morehouse School of Medicine, Atlanta, GA USA; 5 InnerLight Holistic Doula & Perinatal Consulting, Comparative Women’s Studies, Spelman College, Decatur, GA USA; 6 Preeclampsia Foundation, Melbourne, FL USA; 7 Division of Women’s Health, Department of Medicine, Brigham and Women’s Hospital, Boston, MA USA

**Keywords:** Preeclampsia, Hypertensive disorders, Pregnancy, Racial disparities

## Abstract

Hypertensive disorders of pregnancy (HDP) are among the leading causes of maternal mortality in the United States, with Black women and birthing people disproportionately having higher HDP-related deaths and morbidity. In 2020, the Preeclampsia Foundation formed a national Racial Disparities Task Force (RDTF) to identify key recommendations to address issues of racial disparities related to HDP. Recommendations are centered around the Foundation’s three pillars: Community, Healthcare Practice, and Research. Healthcare practices include adequate treatment of chronic hypertension in Black women and birthing people, re-branding low-dose aspirin to prenatal aspirin to facilitate uptake, and innovative models of care that especially focus on postpartum follow-up. A research agenda that examines the influence of social and structural determinants of health (ssDOH) on HDP care, access, and outcomes is essential to addressing disparities. One specific area that requires attention is the development of metrics to evaluate the quality of obstetrical care as it relates to racial disparities in Black women and birthing people with HDP. The recommendations generated by the Preeclampsia Foundation’s RDTF highlight the strategic priorities and are a call to action that requires listening to the voices and experiences of Black women and birthing people, engaging their communities, and multi-sectoral collaboration to improve healthcare practices and drive needed research.

## Introduction

The prevalence of hypertensive disorders of pregnancy (HDP) has been increasing in the United States (US) and is among the leading causes of maternal deaths [1]. Data consistently demonstrates racial disparities in not just the prevalence of HDP but also in maternal and perinatal outcomes [2]. Data from the Centers for Disease Control and Prevention (CDC) Pregnancy Mortality Surveillance System for 2007–2016 show that HDP contributed to a significantly higher proportion of pregnancy-related deaths among Black women than among white women [3]. Black women and birthing people with preeclampsia are particularly at higher risk for severe maternal morbidity, such as stroke, pulmonary edema, and renal failure, and have higher postpartum re-admission rates [4, 5].

Black women and birthing people with HDP carry a higher cardiometabolic burden due to a combination of factors and often enter pregnancy with pre-existing risk factors for cardiovascular disease (CVD), which increases their risk of preeclampsia. Firstly, Black women and birthing people disproportionately experience social, structural, and environmental stressors that are frequently rooted in historic and present-day racism, entrenching them in cycles of marginalization and inequality which can lead to chronic stress. This results in “weathering,” or early health deterioration due to the cumulative impact of repeated experiences with social or economic adversity and political marginalization [6]. Disproportionate physiological deterioration accumulated over time can result in the development of adverse health conditions such as hypertension at an earlier age. In the absence of a direct measure of weathering, allostatic load is often used. Allostatic load is conceptualized as the physiological burden imposed by stress [7]. A recent study found that allostatic load was associated with cardiovascular risk, hypertension, and metabolic disorders 2–7 years postpartum, and that allostatic load was a partial mediator between race and cardiovascular risk. Secondly, Black women and birthing people may be more vulnerable to some of the stress-related behavioral and pathophysiological effects associated with CVD such as poorer dietary and physical activity patterns [8]. Thirdly, Black women and birthing people may be less likely to have their pre-existing medical conditions, and specifically chronic hypertension, adequately managed [9, 10].

## Preeclampsia Foundation’s Approach to Addressing Racial Disparities in Black Women and Birthing People

To recognize and identify key action items and strategies to address issues of racial disparities as they relate to HDP, the Preeclampsia Foundation formed a national Racial Disparities Task Force (RDTF) in May 2020. The objective of the RDTF was to identify the top priorities for the Foundation and all stakeholders to address the racial and ethnic disparities in HDP.

The RDTF included broad representation across the US healthcare industry: nurses, doctors, doulas, researchers, scientists, patient advocacy organizations, and industry and Black, Indigenous, and people of color (BIPOC) women with lived experiences [11]. The facilitators of the RDTF were Preeclampsia Foundation board members, who developed the structure of the task force operations, upheld the overall framework and purpose of the task force, and led the team.

**Fig. 1:**
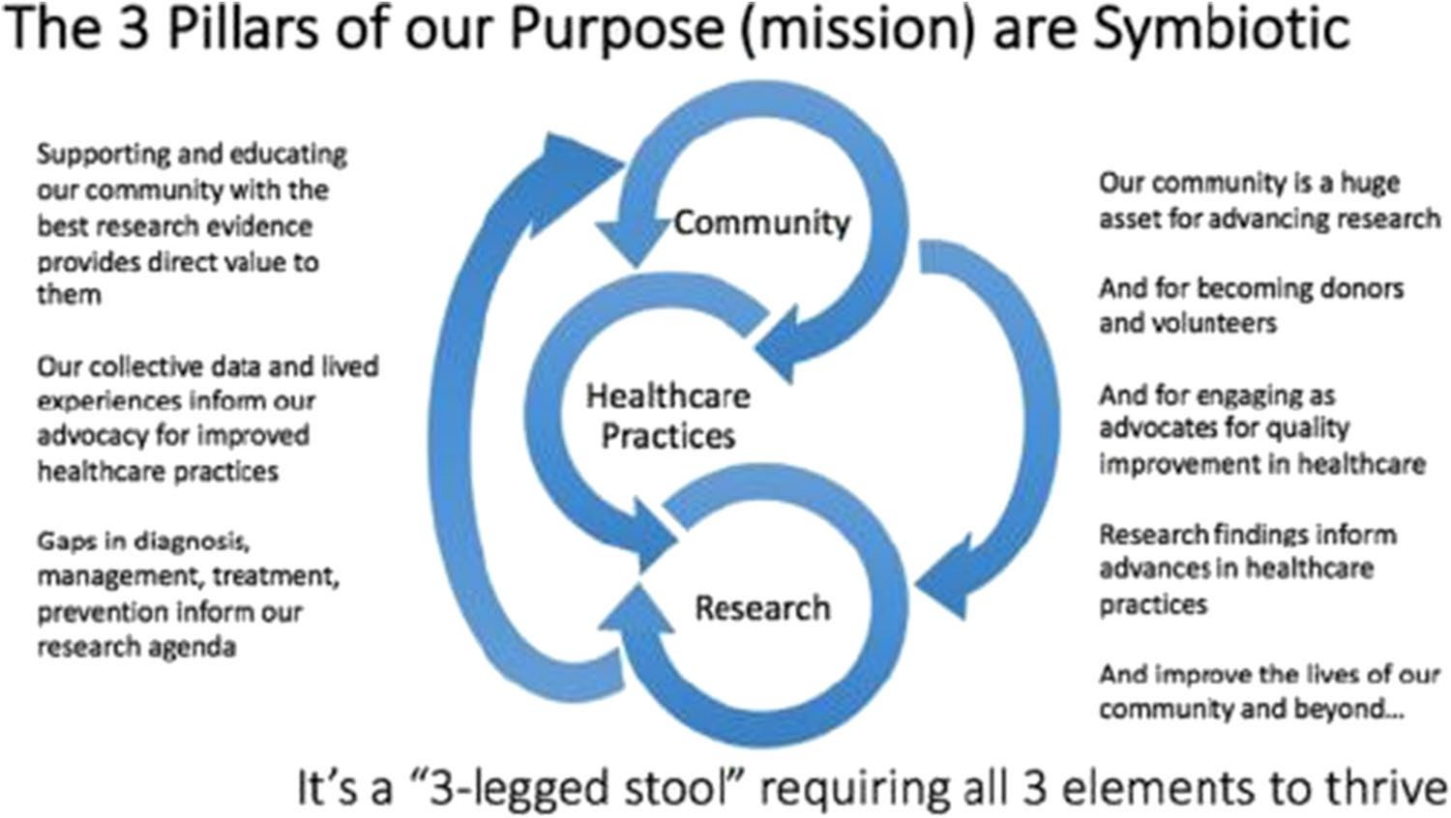
The three pillars of the Preeclampsia Foundation. The Community pillar focuses on patient education, support, and awareness. Healthcare Practices includes clinical providers, payers, state initiatives, and legislative efforts to advance preeclampsia care. The Research pillar aims to find a cure

The RDTF was organized into sub-committees around the Preeclampsia Foundation’s three pillars: Community (e.g., patient education, support, and awareness), Healthcare Practices (i.e., to include clinical providers, payers, state initiatives, and legislative efforts), and Research (i.e., to find a cure). Figure 1 illustrates the Foundation’s three pillars.

Over the course of a year, the full RDTF met monthly with input from guest speakers who had expertise in various areas related to preeclampsia research, clinical management, and lived experience. The sub-committees met more frequently to develop strategies and recommendations related to HDPs under each pillar. The RDTF finalized the set of recommendations through consensus in 2021. The recommendations will be available as a living document, updated as new information becomes available, and are to be written into the Foundation’s strategic and operational plans, as well as serve as a broader call-to-action to the maternal and infant health community.

Although the original goal of the RDTF was to develop recommendations to address racial and ethnic disparities in HDP for BIPOC women and birthing people, committee discussions and recommendations focused on HDP disparities in Black women and birthing people.

In this paper, we discuss the key recommendations within each pillar, with the aim to facilitate a larger call to action to address racial disparities in preeclampsia care affecting Black women and birthing people. The RDTF recognized that many of the recommendations may be complex to implement into existing frameworks and may be resource intensive. However, the hope of the RDTF is that these recommendations will encourage relevant bodies to develop a roadmap for implementation that is contextualized to their setting.

### Community Practices

1. Amplify and center the voices of Black women and birthing people in research and healthcare practice.

Centering the lived experience of Black people enhances the ability to understand their root needs. Engagement with community-based organizations, particularly non-traditional partners outside of mainstream health-serving organizations such as the National Birth Equity Collaborative, Black Mamas Matter Alliance, and Black Women’s Health Initiative, can deepen understanding about the intersections of Black women and birthing people and inform data and strategies to eliminate maternal health inequities.

Researchers and healthcare practices must recognize that Black women and birthing people are not a monolithic group and have diverse experiences and needs. Consideration of subjugations and the multilayered determinants of health (e.g., class, education, gender, race, sexuality, ability) that impact the health of Black women and birthing people are necessary to develop research and provide care that meets their needs [12, 13].

Working in partnership with Black communities and gathering their perspectives must be the initial step toward informing research, healthcare practices, and policy, not an afterthought. Black women and birthing people must be part of the process that amplifies their power at every step and acknowledges their lived experience as expertise. Black women and birthing people, their support networks, and their communities should be actively involved in the co-design and implementation of healthcare trainings. Co-design methods have been shown to have strong and enduring impacts on health [14, 15]. These participatory approaches can tackle the complexity of the issues, aid in inclusivity, help overcome knowledge gaps by valuing lived experience, and shift power dynamics. By providing Black women and birthing people the opportunity to be a partner in their care, they can be empowered to define what they want and need and receive respectful care.

2. Partnering with the community and with community providers.

It is important to build a partnership with community providers because they represent the cultural needs of Black women and birthing people and can promote a model of care that is individualized, respectful, and relationship-based.

Community perinatal professionals such as doulas and community health workers often share the lived and cultural experiences of the patients they serve. When health systems understand this as a strength, they are better equipped to provide patients with essential aspects of holistic care, including cultural congruency, dialogue, reduced unconscious bias, respect for patients’ questions and desires, and a sense of community, as opposed to detached hierarchy. Furthermore, the continuity of care of Black women and birthing people by community perinatal professionals make them the most intimate and trusted advocates on their patients’ birthing team. Their ability to provide informal counseling and guidance on health behaviors and serve as advocates for their patients makes them an important part of the team caring for and working with patients and their families. They can elevate the urgency of addressing hypertension and educate families on more proactive health behaviors in everyday life. By addressing health literacy and social support needs, studies have found that doula services can mitigate the negative health effects of some social and structural determinants of health (ssDOH) during pregnancy and childbirth [16].

Historically in Black communities, community-based outreach programs focusing on hypertension prevention, awareness, treatment, and control have been based in churches, barbershops, and beauty salons [17]. Applying this same format, partnership with local community-based organizations, such as churches or the YMCA, can foster engagement and be a trusted space to host postpartum blood pressure checks and point of care testing, such as HbA1c and lipids for Black patients with HDP.

### Healthcare Practices

1. Optimal management of chronic hypertension.

The management of chronic hypertension in pregnancy in the US has continued to evolve. Until recently, there has been controversy regarding the optimal treatment threshold for chronic hypertension in pregnancy, with previous guidelines suggesting that blood pressure should be treated when it reaches the threshold for severe hypertension, defined as blood pressure greater than 160/110 mmHg [18]. The recently published CHAPS study found that in pregnant women with chronic hypertension, a strategy of targeting a blood pressure of less than 140/90 mmHg rather than reserving treatment only for severe hypertension was associated with better pregnancy outcomes (e.g., lower risk of preeclampsia, fetal growth restriction, and preterm labor), with no increase in the risk of small-for-gestational-age infants [19]. Outside of pregnancy, the lack of treatment intensification in Black patients has been associated with approximately 25% of the observed racial differences in blood pressure control [20]. It is important that chronic hypertension during pregnancy is managed to achieve the treatment target of less than 140/90 mmHg instead of “normalizing” higher blood pressures in Black women and birthing people.

2. Low-dose aspirin.

The US Preventive Task Force for the prevention of preeclampsia and hypertensive complications of pregnancy has recommended the use of low-dose aspirin in pregnancy, as it has been demonstrated in some settings to significantly reduce the risk of preeclampsia and pre-term birth. While a meta-analysis of two trials found variation in the efficacy of aspirin for the prevention of preeclampsia by race and ethnicity, it should be noted that the dose of aspirin used in both trials that were analyzed was 60 mg, which differs from the currently available and recommended low dose of 81 mg in the US [21]. This finding also differs from the secondary analysis of the Aspirin for Evidence-Based Preeclampsia Prevention trial, which found no evidence of heterogeneity in aspirin effect by race and ethnicity [22].

Studies have suggested that inadequate counseling and other factors may contribute to lower rates of aspirin adoption and compliance by Black women compared to all other races [23, 24]. RDTF community members suggest that re-branding low-dose aspirin as “prenatal aspirin” may potentially promote its use, much like the term “prenatal vitamins.” Other barriers to aspirin uptake include access to pharmacies, transportation, and written prescriptions and cultural beliefs about opposing aspirin use in pregnancy.

3. *Postpartum follow-up.*

Although the postpartum period is a critical window to prevent maternal mortality and morbidity and set the stage for long-term health, only 52% of all women and only 47% of Black women with severe preeclampsia attend their 6-week postpartum visit. Black women are less likely to return for an in-person blood pressure check shortly after discharge compared to non-Black women [25]. Emerging data shows higher and more persistent postpartum blood pressure trajectories for Black women [26]. This is particularly concerning because Black patients are less likely to attend a primary care visit in the 12 months following a complicated pregnancy [27].

The RDTF has highlighted four key areas below to optimize the postpartum care of Black women and birthing people.

- Innovative models for postpartum care.

Lessons learned from the implementation of innovative care models that extend and expand care, from text messaging, to doulas, visiting nurses, and community perinatal health workers, and to mobile vans providing care and screening, could be especially important for supporting postpartum care in maternal health deserts with disproportionately high rates of Black patients and those living below the poverty line. These innovative care models that show improved outcomes with support from interdisciplinary teams such as community health workers and care navigators deserve further study [28, 29].

A specific initiative that addresses maternity care deserts in rural areas is the Office of Rural Health Policy RMOMS (Rural Maternity and Obstetrics Management Strategies) initiative.

RMOMS aims to increase access to maternity care in rural communities through four focus areas: obstetrics service aggregation, care coordination along the continuum of care, telehealth and specialty care, and financial sustainability (i.e., payment models). For example, the New Mexico Rural OB Access and Maternal Services (ROAMS) program provides access to obstetric care and telehealth, as well as perinatal and postpartum support, for mothers in rural New Mexico [30]. Additionally, ROAMS provides family navigation to assist with the coordination of services that families need.

b. Home blood pressure monitoring.

There has been growing interest and studies on self-measured blood pressure (SMBP), including novel communication methods such as text messaging and remote Bluetooth wireless data transmission. Patient empowerment to understand blood pressure measurement and management and the need for cardiovascular recovery from their birth experience can benefit their long-term health.

There is data to support home blood pressure monitoring in postpartum patients with HDP [31]. Studies suggest that home blood pressure monitoring decreases the number of visits to the emergency room without compromising care [32]. Remote blood pressure monitoring in the postpartum period has been perceived by patients as easy to use and an acceptable method for monitoring [33]. Another study found that text-based monitoring was more effective in obtaining blood pressure and meeting current clinical guidelines in the immediate post-discharge period in women with pregnancy-related hypertension compared to traditional office-based follow-up [34]. Additionally, text-based monitoring was associated with no racial differences, suggesting that this modality was effective in reducing disparities across different populations [35].

c. Social and structural determinants of health (ssDoH) screening.

Housing, food, transportation, and income security can make it challenging for some women and birthing people to access the care they need and can be a barrier to recovery and appointment attendance for patients recovering from HDP. A recent study found that over 50% of pregnant patients with the highest adverse ssDOH burden had suboptimal cardiovascular health [36]. Implementing routine screening for ssDOH in the clinical setting can be the first step towards identifying resources and overcoming daily challenges.

d. Mental health support.

The postpartum clinical care of patients with preeclampsia often focuses on the immediate physical health issues, but studies have shown that patients can also benefit from mental health screening. Many preeclampsia survivors report their birth experience as traumatic [37]. A recent study that mapped the patient journey through a preeclampsia-complicated pregnancy using data from the Preeclampsia Foundation’s online patient registry found that almost half of the preeclampsia survivors indicated that the experience of having preeclampsia seriously impacted their mental and emotional well-being. 49.3% of survivors reported symptoms of postpartum depression, and 17.3% reported being diagnosed with postpartum depression [38]. Integrating screening for mental health disorders after HDP is necessary and can enable a better postpartum care transition by ensuring that women and birthing people are paired with mental health resources and have reliable follow-up.

### Research

1. Engaging Black women and birthing people, their communities, and the scientific community in research.

There is a critical need for more representative data and thus, the need to involve Black women and birthing people in HDP research, beginning with qualitative studies to understand barriers to engaging women and birthing people in research. The authoritative knowledge that Black women and birthing people have about their lives and health should form the basis of collaboration between researchers and study participants. Healthcare providers can be trusted advocates to encourage their Black patients to participate in research. There is a need for community-based participatory research in the HDP space, including qualitative and mixed methods research, so that investigative and co-creation priorities are set in collaboration with Black women and their communities. It is through the stories of Black women and birthing people and their communities that we will learn about culturally relevant, evidence-based solutions for HDP, as that expertise lies in the lived experience of the people and communities facing HDP inequities. The dissemination of research findings to study participants, and dialogue on those findings, is imperative for the development of sustainable interventions [39].

2. Supporting Black researchers to lead HDP research.

Diversifying research teams will encourage better work on inequities in HDP. Therefore, efforts should be made to support Black researchers to develop and lead this work. Although many Black researchers have the knowledge and expertise, they may feel left out of the leadership team or that they are brought in only in an advisory capacity, feeling marginalized. Valuing the lived experiences of Black researchers and insights, as well as efforts to diversify the research workforce in these spaces, is an important next step. These lived experiences give Black investigators relevant theories and frameworks and can potentially operationalize community-engaged participatory research approaches for the greatest impact [40]. Their perspectives, backgrounds, and lived experiences significantly contribute to what research questions are being asked and how to answer them. Without the experiences and worldviews of Black researchers, the picture is incomplete, and the solutions are inadequate.

3. A research agenda that examines the influence of ssDoH on HDP care, access, and outcomes.

The RDTF has advocated for a shift in the research agenda for HDP management, particularly blood pressure management, towards precision medicine. Precision medicine, sometimes known as “personalized medicine,” is an innovative approach to tailoring disease prevention and treatment that considers differences in patients’ genetics, environment, and lifestyle [41]. It is widely recognized that race is a social and not a biological construct. Simplifying disparities in outcomes to race without deeper examination ignores the fact that relationships between race and health reflect enmeshed social and biologic pathways. Race, therefore, provides a poor proxy for both genetics and precision medicine.

Although outside of pregnancy there are race-based guidelines for blood pressure management, a recent study has shown that race-based prescribing is ineffective, unwarranted, and may even be detrimental to the long-term health of Black patients [42]. Observational work during pregnancy has shown that race alone is a poor predictor of response to certain anti-hypertensive medications, while the consideration of maternal volume status and hemodynamic profiles may prove to be promising [43].

The research agenda in HDP should focus on targeted interventions that address the hemodynamics and ssDoH of individual Black women and birthing people so that we can work towards precise and individualized therapy. As data suggests that low-dose aspirin may not be as effective in Black women and birthing people, there should be further investigation to determine optimal dosing adherence strategies and alternative therapies [21]. Further work is needed in this space, especially on the nuances of medication and medication combination efficacy, medication side effect profiles, and daily dosing schedules to optimize hypertension management, where there has been limited research [44].

4. Obstetrical quality of care and racial disparities.

There is a paucity of literature on obstetrical quality of care and racial disparities in maternal and perinatal outcomes related to HDP. A growing body of research is now focusing on racism, discrimination, and bias in maternal healthcare and their association with lower quality of care delivery [45–47]. A first step would be to identify quality measures relevant to HDP and create a performance dashboard at an institutional level that disaggregates data by race and ethnicity to better understand where disparities exist. Emphasis also needs to be placed on quality measures that are meaningful to Black women and birthing people.

Recently, the Society for Maternal–Fetal Medicine proposed a uniform metric reflecting the rate of timely postpartum follow-up of patients with severe hypertension [48]. A recent study has shown that timely treatment of severe hypertension significantly improved with the implementation of targeted quality measures [49]. An accompanying quality measure would examine how protocols and standards of care related to the identification and management of severe hypertension (i.e., 160/110 mmHg) are being applied to Black women and birthing people during pregnancy and the postpartum period. Studies have found that the implementation of a semiautonomous treatment algorithm for severe hypertension was associated with a higher percentage of pregnant and postpartum patients receiving the first dose of antihypertensive therapy within 15 and 30 min [50].

**Box 1** Research and improvement gaps

**Table:** 

• High-quality and reliable data on the national incidence of pre-eclampsia and rates of related complications by race/ethnicity, especially in maternity care deserts
• Quality of care in non-English speaking Black women and birthing people with HDP as well as Black patients with HDP in maternity care deserts
• Use data from state maternal mortality review committees to determine the practice gaps that need to be addressed locally to provide better care to Black women and birthing people with HDP
• Impact of innovative models of care (e.g., mobile van visits, virtual clinics, home visiting programs, remote blood pressure monitoring) on maternal and perinatal outcomes in Black women and birthing people
• Involvement of community perinatal professionals on maternal and perinatal outcomes in Black women and birthing people
• Examine healthcare bias on aspirin initiation in Black women and birthing people and exploration of the factors that influence aspirin acceptability and uptake
• Use hemodynamic profiles to develop a better understanding of how to select effective antihypertensive therapy for different groups of Black women and birthing people
• Studies to inform guidelines for timing of postpartum cardio-metabolic screening and interventions specifically tailored to different groups of Black women and birthing people
• Exploration of the barriers and facilitators to postpartum follow-up after HDP in Black women and birthing people
• Short- and long-term mental health and the psychosocial impact of HDP in Black women and birthing people and interventions (e.g., group sessions) to address these issues

### Considerations for Implementation

1. Community practices.

Implementation of a community-based model can be challenging. However, a participatory model can be developed that focuses on using the community as a stakeholder to identify and express the specific needs of the community. Follow-up for hypertensive complications is especially important in the postpartum period. A systematic review and meta-analysis of strategies in high-income countries and their impact on antepartum and postpartum care found that patients who received enhanced support with individualized care were significantly more likely to attend their postpartum visit [51].

Strategies for consideration to improve follow-up for women and birthing people at risk of hypertensive complications which have shown promise include patient navigators, who work in the community and can schedule follow-up visits, and patient incentives [29]. The expanding technology field has not been fully utilized to reach birthing populations. The use of artificial intelligence (AI) to diagnose preeclampsia may increase healthcare access and availability to patients with barriers to care [52].

Further studies to find optimal strategies through a culturally sensitive lens with a concentration on equity are needed. Additionally, developing a reimbursement model for holistic, comprehensive care should be part of long-term planning, as preliminary evaluations have demonstrated significant improvement in maternal and fetal outcomes using an integrated model [53].

2. Healthcare practices.

To effectively implement the proposed recommendations for healthcare practices, community involvement, muti-disciplinary collaboration, and coordination between organizations working in the space of HDP are necessary. For example, effectively promoting aspirin as “prenatal aspirin” will require uniform messaging across organizations. Further work with the community is also needed to evaluate identify and address barriers regarding aspirin acceptability and uptake by Black women and birthing people.

Effectively implementing some recommendations, such as making blood pressure monitors and appropriately sized cuffs available and accessible, will need multiple challenges to be addressed. Every state has different policies, and these devices are often not covered by public insurance or third-party payers. Even if covered, the monitors may need to be collected from a medical supply store instead of a usual pharmacy where patients collect their medications. In response, the Preeclampsia Foundation initiated the Cuff Kit program to facilitate this process by sourcing pregnancy-validated monitors and producing and including patient education materials to fill this need [54].

Perhaps one of the biggest challenges is the need for a paradigm shift to focus on pre-conception and postpartum blood pressure optimization. Involving and educating primary care physicians is key to effectively implementing these recommendations. Addressing issues such as the ssDOH and mental health in postpartum care requires a multi-disciplinary team. Real-life challenges include the lack of mental health providers trained to care for postpartum women and birthing people and the lack of funding for obstetric navigators and social workers.

3. Research practices.

Individual researchers must acknowledge the impact of systemic racism on health, engage in self-education to mitigate biases, hire diverse teams, and include communities historically excluded in research. Institutions must provide clear guidelines on the use of race and ethnicity in research, reject stigmatizing language, and invest in systemic commitments to diversity, equity, and anti-racism [55]. National organizations must call for race-conscious research standards and training and create measures to ensure accountability. A cross-sectional study noted that, while the number of principal investigators with more than three grants increased three-fold between 1991 and 2020, female and Black principal investigators were under-represented in groups with more than three NIH grants. Not only were Black women underrepresented in these groups, but they were also 70% less likely to obtain super principal investigator status than white men. Mentorship of those underrepresented in research needs to be a specific focus to implement the changes proposed in this paper. Women, especially Black women, are less likely to receive early career mentorship than white men and are more likely to be given institutional tasks that do not lead to promotion.

One way to achieve success is to expand funding for racially diverse teams, as data suggest these teams are more innovative and productive [55]. In 2021, the NIH instituted the UNITE project which has sought to address structural barriers and has made progress in improving the diversity of the research workforce [56, 57]. While increasing Black researchers can help with enrollment, a recent trial demonstrated that Black patients are likely to and want to participate in clinical research when barriers are fully addressed [58].

## Conclusion

The recommendations generated by the Preeclampsia Foundation’s RDTF highlight strategic priorities, for which this paper is a call to action. The core component of effectively implementing these recommendations and addressing racial disparities in HDP is listening to the voices and lived experiences of Black women, birthing people, and their communities, as well as engaging and including them in research.

To address racial disparities in the care of Black patients with HDP, pregnancy needs to be situated within a continuum and should be viewed as an entry point for preventative care.

Postpartum care and long-term follow-up of Black women and birthing people with HDP remains a critical gap for which innovative approaches need consideration.

Optimizing current models of care requires a multi-sectoral approach that incorporates the ssDOH and mental health, as many Black women and birthing people live at the intersection of other marginalized identities. While in the short term, we can work on delivering contextualized and tailored clinical care, our long-term goal should be to ensure that our organizational policies and clinical guidelines reflect the needs of Black women and birthing people with HDP.
